# Mass Spectrometry Analysis Coupled with *de novo* Sequencing Reveals Amino Acid Substitutions in Nucleocapsid Protein from Influenza A Virus

**DOI:** 10.3390/ijms15022465

**Published:** 2014-02-11

**Authors:** Zijian Li, Wanchun Sun, Donglin Wu, Xiang Gao, Ningning Sun, Ning Liu

**Affiliations:** 1 Key Laboratory of Cardiovascular Molecular Biology and Regulatory Peptides, Ministry of Health/Key Laboratory of Molecular Cardiovascular Sciences, Ministry of Education/Beijing Key Laboratory of Cardiovascular Receptors Research, Institute of Vascular Medicine, Peking University Third Hospital, Beijing 100191, China; E-Mail: lizijian@bjmu.edu.cn; 2 Key Laboratory of Zoonosis, Ministry of Education, Jilin University, Changchun 130062, Jilin, China; E-Mail: Wanchunsun@jlu.edu.cn; 3 Center for Disease Control and Prevention, Changchun 130025, Jilin, China; E-Mail: dl_wu@163.com; 4 Central Laboratory, Jilin University Second Hospital, Changchun 130041, Jilin, China; E-Mails: gaoxiang13@mails.jlu.edu.cn (X.G.); ning668jing@126.com (N.S.)

**Keywords:** nucleocapsid protein, influenza virus A virus, amino acid substitution, mass spectrometry

## Abstract

Amino acid substitutions in influenza A virus are the main reasons for both antigenic shift and virulence change, which result from non-synonymous mutations in the viral genome. Nucleocapsid protein (NP), one of the major structural proteins of influenza virus, is responsible for regulation of viral RNA synthesis and replication. In this report we used LC-MS/MS to analyze tryptic digestion of nucleocapsid protein of influenza virus (A/Puerto Rico/8/1934 H1N1), which was isolated and purified by SDS poly-acrylamide gel electrophoresis. Thus, LC-MS/MS analyses, coupled with manual *de novo* sequencing, allowed the determination of three substituted amino acid residues *R452K*, *T423A* and *N430T* in two tryptic peptides. The obtained results provided experimental evidence that amino acid substitutions resulted from non-synonymous gene mutations could be directly characterized by mass spectrometry in proteins of RNA viruses such as influenza A virus.

## 1. Introduction

Influenza virus has long been a global health threat since 1918 [1]. Although the annually circulating strains of influenza virus are not very virulent, there is still concern that the genome of these seasonal virus strains can mutate to acquire the ability to cause mortality in humans [2,3]. Additionally, for the avian influenza virus strains that usually show no adaptation to a human host, the virus genome can mutate to allow the virus to cross the species barrier to infect humans. For example, H5N1, H1N1 and recently reported H7N9 virus strains have shown their ability to cause severe infections in humans [4–6].

Mutations in viral genomes, some of which are non-synonymous mutations and thus result in amino acid substitutions, are often detected by gene sequencing [7–9]. With the introduction of soft ionization techniques such as ESI and MALDI, characterization of large biomolecules such as proteins has been achieved with high sensitivity and accuracy. Mass spectrometry has been used to analyze several mutations in hemoglobin variants [10,11]. Up to seven amino acid substitutions in HA of influenza A virus were revealed by mass spectrometry [12].

As influenza A virus has a relatively high mutation rate, there will always be an urgent need to detect variation in amino acid sequences resulting from non-synonymous SNPs that may have functional consequences. While both DNA and RNA have served as targets for most genotyping screen strategies, the other major functional molecule, protein, has recently been explored as a source for proteotyping, wherein a variety of protein forms from a single gene are characterized through sophisticated mass spectrometric techniques [13]. Similar to DNA/RNA-based genotyping, proteotyping strategy can be applied on either a single protein [14] or on a proteome-wide scale [15]. Because influenza A virus continues to mutate to evolve, the previously established DNA/RNA-based PCR approaches often fail to detect the newly emerging strains due to sequence variation in primer and probe [16]. However, for the protein-based proteotyping strategy, the mutated peptides or modified peptides van be detected without the concerns in PCR approaches. Therefore, once the proteotyping strategy is optimized for any given strain, it should be effective to detect an array of isoforms of viral proteins, including the peptides upon modification and amino acid substitution. In this study, we report the characterization of the nucleocapsid protein (isolated and purified by SDS-PAGE) of influenza A virus by mass spectrometry. By manual interpretation of the MS/MS data, three amino acid substitutions were identified. The results indicated that mass spectrometry coupled with *de novo* peptide sequencing had the power to characterize the amino acid substitutions in proteins of RNA viruses such as influenza A virus.

## 2. Results and Discussion

### 2.1. Identification of NP Protein

**Figure 1.**
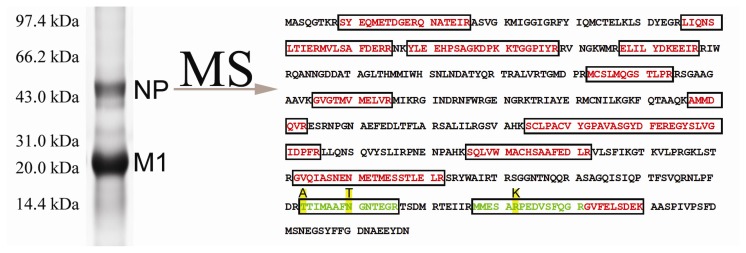
Identification of nucleocapsid protein from purified influenza virus (A/Puerto Rico/8/1934 H1N1). The purified virus was lysed and separated on 12% SDS-PAGE. The upper band around 56 kDa was cut off and subjected to in-gel digestion, followed by mass spectrometric analysis. Database searching identified 18 tryptic peptides (labeled with red) from nucleocapsid protein of influenza A virus. Manual interpretation of the obtained MS/MS data identified three amino acid substitutions (*R452K*, *T423A* and *N430T*, highlighted with yellow) within two tryptic peptides (labeled with green).

Influenza virus was inoculated in chick embryos. Several serial passages were performed to enhance the rates of multigenic mutations. The virus particles were purified from the collected allantoic fluid and then lysed and separated on 12% SDS-PAGE. After staining with Colloidal Coomassie G250, two major bands were found at 15 and 56 kDa, respectively (Figure 1). The band at 56 kDa was cut off and subject to in-gel tryptic digestion. LC-MS/MS analysis of the obtained peptide mixture coupled with protein database searching identified a total of 18 unique peptides of nucleocapsid protein from influenza virus (A/Puerto Rico/8/1934 H1N1) (Table 1).

Besides the peptides identified by database searching, two additional mutated peptides were determined by manual interpretation of the available data, in which three amino acid substitutions were identified. Accordingly, both database searching and manual interpretation of the obtained LC-MS/MS data allowed the assignment of a total of 20 unique peptide sequences.

### 2.2. Identification of AA Substitution of R452K

**Figure 2.**
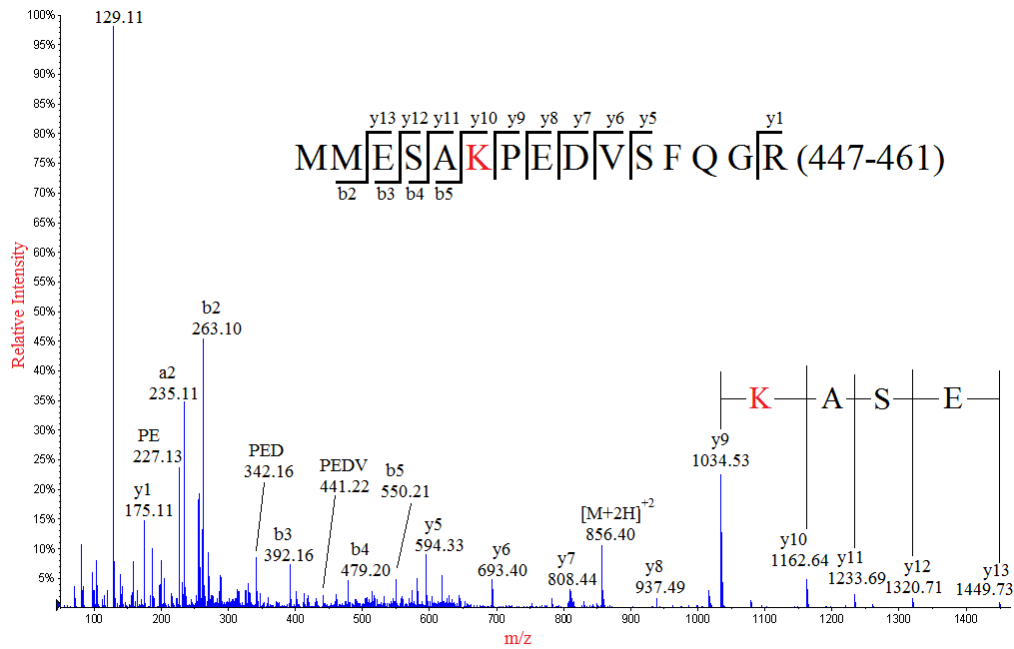
MS/MS spectrum of the doubly-charged ion at *m*/*z* 856.40 from the analysis of peak MP1. The mutated peptide (MMESAKPEDVSFQGR) of a normal sequence (residues 447–461) from tryptic digestion of nucleocapsid protein was identified, in which the R452 was substituted with K.

Interpretation of the MS/MS spectrum of the doubly-charged ion peak MP1 at *m*/*z* 856.40 (Figure 2) allowed the identification of a partial sequence of ESA, considering the ion series of *m*/*z* 1449.73, 1320.71, 1233.69, 1162.64 at the high mass end of the spectrum were *y* type fragment ions *y*13, *y*12, *y*11, *y*10, respectively. The sequence of ESA was readily to be located in one of the tryptic peptides of NP: MMESARPEDVSFQGR (447–461) with theoretical *m*/*z* value of 870.40 for its doubly-charged ion. Thus, a nominal mass shift of −28 Da was observed for the detected doubly-charged ion of MP1 in comparison with the molecular weight of the theoretical sequence of MMESARPEDVSFQGR (447–461) in NP, which might result from amino acid substitution of one of five residues in the theoretical sequence: R→Q/K, V→A, M→C, D→S or E→T. The possibility for amino acid substitution of M→C was readily eliminated because the Cys (C) residue would be chemically alkylated during sample preparation if Methionine (M_447/448_) was mutated into Cys (C). Noticeably, the fragment ion *y*9 at *m*/*z* 1034.53 adjacent to *y*10 ion (*m*/*z* 1162.64) in the high mass range of the MS/MS spectrum indicated that the residue next to the Alanine (A_451_) should be either K or Q, considering that the calculated difference between 1162.64 and 1034.53 was identical to the nominal mass of 128 of either of these two amino acid residues. In addition, substitution of Arginine (R_452_) with either Lysine (K) or Glutamine (Q) was also confirmed by the detection of the base peak at *m*/*z* 129, which was the immonium ion of either K or Q. Although both K and Q residues had identical nominal mass of 128, the exact masses of them were different (K with 128.095 and Q with 128.058). The precise mass difference between *y*9 and *y*10 was calculated as 128.11, suggesting that the R_452_ was substituted by K, but not Q. This conclusion was well supported by the precise mass data of the immonium ion detected at *m*/*z* 129.11, which was much closer to the theoretic mass data of immonium ion of K (129.1022) than that of Q (129.0659). The assignments of most *y* series ions (from *y*5 to *y*13) clearly demonstrated the internal sequence of PEDV, eliminating the possibilities of amino acid substitutions at E_454_, D_455_ and V_456_. It should be noticed that there was a Proline (P) in the sequence, at which internal fragmentation could occur. Some internal sequences such as PE, PED, PEDV and PEDVS were detected and assigned, confirming that amino acid substitution should occur at R_452_ but not E_454_, D_455_ and V_456_. Additionally, detection of some of the *a* and *b* series ions such as *a*2, *a*3, *b*2 and *b*3 indicated that E_449_ was not subject to amino acid substitution, confirming the substitution of R_452_→K.

### 2.3. Identification of AA Substitution T423A and N430T

**Figure 3.**
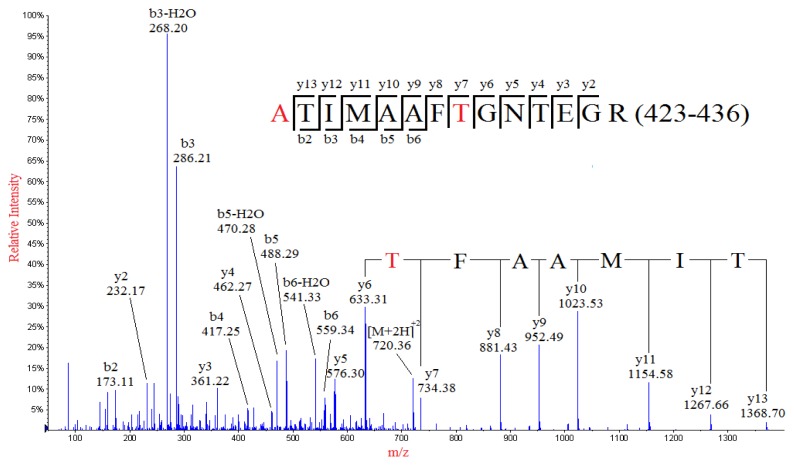
MS/MS spectrum of the doubly-charged ion at *m*/*z* 720.36 from the analysis of peak MP2. The mutated peptide (ATIMAAFTGNTEGR) of a normal sequence (residues 423–436) from tryptic digestion of nucleocapsid protein was identified, in which the T423 and N430 were substituted with A and T, respectively.

*De novo* sequencing of the MS/MS spectrum of a doubly-charged ion peak MP2 at *m*/*z* 720.36 identified a partial sequence of TIMAAFT with *y* series ions at *m*/*z* 1368.68, 1267.71, 1154.61, 1023.56, 952.53, 881.46, 734.42 and 633.35 (Figure 3), which was not found in the theoretical sequence of NP. However, investigation of the theoretical sequence of NP revealed a sequence of TIMAAFN (424–430), which was identical to the deduced sequence except for the N_430_ residue. Therefore amino acid substitution of N_430_→T was identified, which resulted in a mass shift of −13.01 Da. The identified sequence was contained in a tryptic peptide of NP: TTIMAAFNGNTEGR (423–436), of which the calculated *m*/*z* value of the doubly-charged ion was 741.86. However, a nominal mass shift of −43 but not −13.01 Da (N_430_→T) was observed for MP2 when compared to the theoretical sequence of TTIMAAFNGNTEGR (423–436), suggesting that there might be at least one additional amino acid substitution in the sequence, which resulted in an additional mass shift of −30.01 Da. Investigation of the rest of the residues of the tryptic peptide sequence (423–436) indicated that there were three amino acid residues that could result in a mass shift of −30.01 Da upon substitution: T_423_→A, T_433_→A, E_434_→T. The detection of the *y* series ion *y*13 at *m*/*z* 1368.66, as well as *b*_3_-H_2_O, *b*_3_, *b*_2_-H_2_O and *b*_2_ ions, indicated that the first three residues in the peptide were ATI, thus confirming the identification of substitution of T_423_→A. Therefore, the peak MP2 was identified as the tryptic peptide in the residues from 423 to 436 with the two substitutions, namely T_423_→A and N_430_→T.

### 2.4. Bioinformatics Analysis

**Figure 4.**
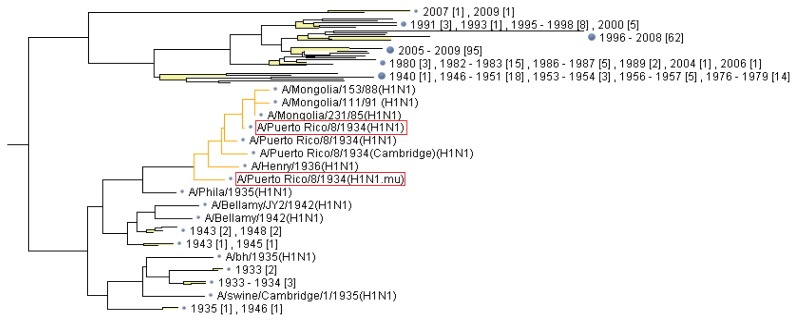
Part of detailed tree picture generated from linkage analysis of mutated nucleocapsid protein identified by MS/MS, by which the neighbor-joining method of clustering was used. The strain containing the mutated nucleocapsid protein (A/Puerto Rico/8/1934 (H1N1.mu)), as well as its original strain (A/Puerto Rico/8/1934 (H1N1)), is labeled with red rectangular box.

The sequences of nucleocapsid proteins were exclusively retrieved from “The FLU project” at GenBank. A protein sequence database containing the retrieved sequences and the mutated sequence was built and subject to multiple alignment and linkage tree analysis (Figure 4). The output file containing the whole tree data can be found in supplemental materials.

## 3. Experimental Section

### 3.1. Chemicals and Materials

Sequencing-grade TPCK-modified trypsin was purchased from Promega (Madison, WI, USA). Bradford protein assay kit, ammonium bicarbonate, dithiothreitol (DTT), iodoacetamide (IAA) were purchased from Bio-Rad (Hercules, CA, USA). All the other chemicals were purchased from Sigma-Aldrich (St. Louis, MO, USA). Influenza virus (A/Puerto Rico/8/1934 H1N1) was propagated in a biosafety level 2 (BL-2) containment facilities. Ultra-pure water was prepared by a MilliQ water purification system (Millpore, Bedford, MA, USA).

### 3.2. Virus Cultivation and Purification

Embryonated chicken eggs were inoculated with the influenza A virus (A/Puerto Rico/8/1934 H1N1) and incubated for 72 h at 37 °C. The allantoic fluid was harvested, followed by centrifugation at 5000 rpm for 15 min. The virus in the allantoic fluid was pelleted through a 4-step discontinuous gradient cushion consisting of 30%, 40%, 50% and 60% (*w*/*v*) sucrose, in a SW40 Ti rotor (Beckman-coulter, Fullerton, CA, USA) at 35,000 rpm at 4 °C for 60 min. The virus band between 40% and 50% sucrose was carefully collected, and suspended in 10 mM Tris-HCl pH 8.0, 150 mM NaCl. Aliquots of the purified virus sample were kept at 4 °C.

### 3.3. SDS-PAGE

The purified virus particles were lysed with 2× Laemmli sample buffer and kept at 95 °C for 5 min. The protein concentration was assayed with Micro BCA (bicinchoninic acid) protein assay kit (Pierce, Rockford, IL, USA). Electrophoretic separation was performed in a Mini-Cell system (Bio-Rad, Hercules, CA, USA), and run in 12% tris-glycine-SDS polyacrylamide gels with a 5% stacking gel. After electrophoresis, the gels were stained with colloidal Coomassie G250 and scanned with a calibrated densitometer (GS800, Bio-Rad).

### 3.4. In-Gel Digestion

Protein bands of interest were cut off from gels and washed with Milli-Q water three times. Then the gel pieces were destained with a solution of 50 mM NH_4_HCO_3_ in 50% ACN until the Coomassie blue in the gel became invisible. The destained gel pieces were reduced in 10 mM DTT, 50 mM NH_4_HCO_3_ aqueous solution at 60 °C for 60 min, followed by alkylation in 50 mM IAA, 50 mM NH_4_HCO_3_ aqueous solution at room temperature in dark for 30 min. The gel pieces were dehydrated with ACN, and then incubated in freshly prepared digestion solution containing 50 mM NH_4_HCO_3_ and 0.1 g/L TPCK-trypsin overnight at 37 °C. The resulting tryptic peptides were extracted with 5% trifluoroacetic acid (TFA) in 60% ACN and stored at −20 °C until LC-MS/MS analysis.

### 3.5. Capillary LC-MS/MS Analysis

The tryptic peptides were lyophilized and redissolved in high performance liquid chromatography (HPLC) buffer A (0.1% formic acid) and then separated on a C18 column (100 mm × 180 μm i.d.). The elution gradient was from 5% to 40% buffer B (0.1% formic acid, 99% ACN, flow rate: 0.2 μL/min) for 90 min. The eluted peptides were then analyzed on an ABI QSTAR spectrometer using information dependent acquisition mode (IDA; Analyst QS, Applied Biosystems, Carlsbad, CA, USA) by selecting the three most intense ions for MS/MS analysis. A survey scan of 300–2000 Da was collected for 3 s followed by 5 s MS/MS scans of 40–1500 Da using the standard rolling collision energy settings. The dynamic exclusion time was set as 1.5 min.

MASCOT generic files were generated from the obtained MS data by using a script embedded in the Analyst QS 2.0 software (MDS Sciex, South San Francisco, CA, USA) and used to search against the Swiss-Prot protein database on a local MASCOT server (version 2.1, Matrix Science, London, UK). One missed cleavage was allowed. Carbamidomethylation of cysteines was specified as fixed modification, whereas oxidation of methionine was selected as variable modification. The mass tolerance was set to 0.3 and 0.6 Da for peptide and MS/MS ion masses, respectively. Manual *de novo* sequencing of peptide tandem mass spectra was performed with the aid of Pepsea (1.1) in Analyst QS 2.0 software (MDS Sciex).

### 3.6. Bioinformatics Analysis

The mutated nucleocapsid protein containing three amino acid substitutions was analyzed by using a suite of bioinformatics tools at NCBI (http://www.ncbi.nlm.nih.gov/genomes/FLU/FLU.html) [17].

## 4. Conclusions

We herein identified by LC-MS/MS analysis three amino acid substitutions in nucleocapsid protein from influenza virus (A/Puerto Rico/8/1934 H1N1). The three amino acid substitutions were located in two tryptic peptides of the nucleocapsid protein. One of identified amino acid substitutions, *R452K*, was located within the tryptic peptide MP1 (447–461), whereas the other two amino acid substitutions, *T423A* and *N430T*, were located within tryptic peptide MP2 (423–436). Both of the peptides were identified through manual interpretation of the relating MS/MS data, which included both calculation of high resolution MS data and assignment of fragment ions in MS/MS data. The outcome of this study indicated that the MS/MS analysis of amino acid substitutions might be useful in investigating the antigens from influenza viruses.

## Figures and Tables

**Table 1.** Summary of tryptic peptides identified in nucleocapsid protein from influenza virus (A/Puerto Rico/8/1934 H1N1) by database searching.

Peptide No.	Peptide sequence	Charge status	Calculated *m*/*z* (monoisotopic)	Measured *m*/*z* (monoisotopic)	Residues
P1	TGGPIYR	2	382.21	382.21	92–98
P2	KTGGPIYR	2	446.26	446.29	91–98
P3	AMMDQVR	2	441.69	441.70	237–243
P4	QNATEIR	2	416.22	416.22	20–26
P5	GVFELSDEK	2	512.25	512.26	462–470
P6	MVLSAFDER	2	534.26	534.27	66–74
P7	YLEEHPSAGK	2	565.78	565.78	78–87
P8	YLEEHPSAGKDPK	2	735.86	735.86	78–90
P9	LIQNSLTIER	2	593.84	593.85	56–65
P10	GVGTMVMELVR	2	612.31	612.32	185–195
P11	MVLSAFDERR	2	620.31	620.31	66–75
P12	EGYSLVGIDPFR	2	676.85	676.85	294–305
P13	MCSLMQGSTLPR	2	706.82	706.82	163–174
P14	SYEQMETDGER	2	680.77	680.77	9–19
P15	ELILYDKEEIR	2	710.89	710.89	107–117
P16	GVQIASNENMETMESSTLELR	2	1170.05	1170.07	362–382
P17	SCLPACVYGPAVASGYDFER	2	1110.00	1110.00	274–293
P18	SQLVWMACHSAAFEDLR	2	1010.97	1010.98	326–342

M: mono-oxidized methionine.
